# Nanofiber Composites of Poly(vinyl alcohol)/Silver-Based Molybdate and Tungstate Oxide Semiconductors for Antimicrobial Applications

**DOI:** 10.1021/acsomega.4c07471

**Published:** 2025-01-15

**Authors:** Vicente de Sousa Marques, Lee Marx Gomes de Carvalho, Débora Aparecida de Almeida, Rian Richard Santos de Farias, Andressa Dalolio Valente, Alessandro Francisco Martins, Celso Nakamura, Edvani Curti Muniz

**Affiliations:** † Department of Chemistry, Federal University of Piauí (UFPI), Teresina, PI 64049-550, Brazil; ‡ Department of Chemistry, State University of Maringá (UEM), Maringá, PR 87020-900, Brazil; § Department of Chemistry, Pittsburgh State University (PSU), Pittsburgh, Kansas 66762, United States

## Abstract

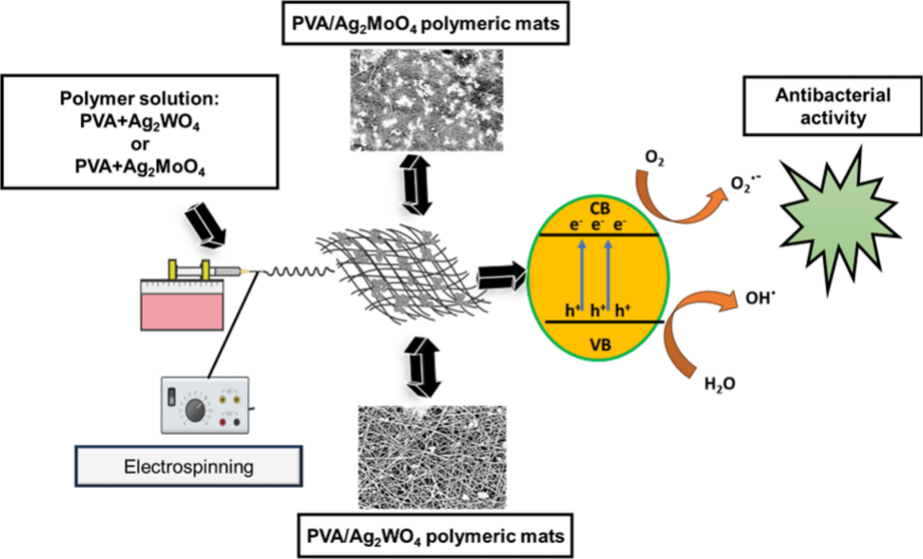

In the present study, powders of α-Ag_2_WO_4_ (PAW) and β-Ag_2_MoO_4_ (PAM) were prepared through the coprecipitation method, while poly(vinyl alcohol) nanofibers (FPVA) and composite nanofibers of PVA/α-Ag_2_WO_4_ (FPAW) and PVA/β-Ag_2_MoO_4_ (FPAM) were prepared using the electrospinning technique. Several characterization techniques were applied to evaluate the structure of the obtained materials, as well as studies for assessing their antimicrobial properties. The antimicrobial activities of the composites against *Pseudomonas aeruginosa* and *Staphylococcus aureus* were investigated through the minimum inhibitory concentration (MIC) and the minimum bactericidal concentration (MBC). Our studies demonstrated that materials exhibit antibacterial activity against *P. aeruginosa* (MIC/MBC = 0.014/ND mg mL^–1^ for PAW; MIC/MBC = 1.43/1.43 for PAM; MIC/MBC = 1.35/1.35 mg mL^–1^ for FPAW; MIC/MBC = 3.68/11.03 mg mL^–1^ for FPAM and MIC/MBC = 8.78/ND mg mL^–1^ for FPVA) and *S. aureus* (MIC/MBC = 0.794/ND mg mL^–1^ for PAW; MIC/MBC = 1.43/ND for PAM; MIC/MBC = 1.35/1.35 mg mL^–1^ for FPAW; MIC/MBC = 3.68/3.67 mg mL^–1^ FPAM and MIC/MBC = 14.63/* mg mL^–1^ for FPVA). The cytotoxic concentrations (CC_50_, μg mL^–1^) against the VERO cells were 21.74 ± 0.04 for PAW, <15 for PAM, 103.70 ± 18.90 for FPAW, 111.22 ± 4.02 for FPAM, and >1000 for FPVA, thus indicating that the immobilization of the semiconductor to the FPVA mats decreases the cytotoxic effect of the materials studied as compared to not immobilized ones. The results suggest that powders and composite polymeric mats displayed antimicrobial action that was attributed to the production of reactive oxygen species (ROS), which are responsible for inducing high local oxidative stress, causing the death of both types of bacteria.

## 1 Introduction

In a global context, developing new technological materials with unique properties has attracted significant attention from the scientific community, usually leading to the development of advanced technologies.^1^ Due to the many environmental problems caused by pathogenic microorganisms, public health has been threatened by disposing unwanted chemicals.^1^ As a result, there is an increasing focus on developing eco-friendly and sustainable materials.^1,2^

Silver molybdate (Ag_2_MoO_4_) and silver tungstate (Ag_2_WO_4_) have attracted considerable attention in the field of antimicrobial research due to their remarkable properties.^3^ These compounds combine the antimicrobial action of silver ions with the unique catalytic properties of molybdate and tungstate anions, resulting in materials with potential applications in various fields, including medicine, agriculture, and water treatment.^3,4^

Silver, in its various forms, has been known for its antimicrobial properties for centuries.^5^ Silver ions (Ag^+^) interfere with the cell membrane of microorganisms, altering permeability and causing the release of essential cellular components, eventually leading to cell death. Additionally, silver ions can bind to bacterial enzymes and proteins, deactivating their vital functions.^5^

When silver ions are combined with molybdate or tungstate anions, the antimicrobial efficacy can be further enhanced. Silver molybdate (Ag_2_MoO_4_) exhibits photoinduced properties that generate reactive oxygen species (ROS) when exposed to light. These ROS are highly reactive and can damage vital cellular components such as nucleic acids, proteins, and lipids, resulting in the death of microorganisms.^6^

Silver tungstate (Ag_2_WO_4_) generally exhibits a higher ROS generation potential compared to silver molybdate (Ag_2_MoO_4_).^7^ This is because Ag_2_WO_4_ has a wider band gap, which allows it to absorb higher energy photons, promoting the excitation of electrons to the conduction band.^8^ The efficient separation of electron–hole pairs in Ag_2_WO_4_ enables more prolonged interactions with water and oxygen molecules, facilitating the formation of ROS like hydroxyl radicals (^•^OH) and superoxide anions (O_2_^•–^).^8,9^ In contrast, Ag_2_MoO_4_, with its narrower band gap, might generate fewer ROS under similar conditions, although it can absorb light over a broader range, including the visible spectrum.^8−10^ This gives to Ag_2_MoO_4_ a slight advantage in applications requiring visible light photocatalysis but may limit its ROS generation potential relative to Ag_2_WO_4_ under UV light.^8^

The active sites in these materials, where electron–hole separation and ROS generation occur, are critical for their antibacterial properties. Ag_2_WO_4_ tends to have more exposed and efficient active sites due to its unique crystal structure, which favors surface reactions and the generation of ROS.^11,12^ On the other hand, Ag_2_MoO_4_ also has effective active sites, but they may not be as abundant or as efficient in facilitating ROS generation as those in Ag_2_WO_4_.^13^ Tungsten has a stronger ability to induce oxygen vacancies and defects, which are key to enhancing ROS generation.^12^

Several methods for obtaining silver tungstate and silver molybdate are cited in the literature, such as solvothermal method,^14,15^ hydrothermal synthesis,^12,16,17^ coprecipitation method,^18,19^ and sonochemistry.^20,21^ The coprecipitation method is notable for its rapid processing, low cost, ability to wash and remove soluble impurities before calcination, and reduced mass loss compared to other methods.^19^

By preparing metal oxides through the coprecipitation method and subsequently incorporating them into polymeric matrices, it is possible to obtain nanocomposite heterostructures that exhibit new properties.^22^ Thus, combining the coprecipitation with the electrospinning technique is a suitable way to obtain these types of materials.^22^ Electrospinning is a process that uses electrostatic forces to create polymeric fibers with diameters typically on the micrometer or nanometer scale.

Among the various polymers used in electrospinning, poly(vinyl alcohol) (PVA) stands out for its solubility in water,^23,24^ excellent film-forming properties,^25^ and chemical inertness and biocompatibility.^25,26^ PVA is widely used in the preparation of polymeric nanofibers, and in many studies, PVA is associated with metal oxides or semiconductors in the preparation of composite nanofibers or polymeric mats.^27^ Once associated with the polymer matrix, oxides can act as catalysts or photocatalysts for various chemical reactions and are also used in drug delivery systems, tissue engineering structures, and antibacterial coatings.^27,28^

Based on the above statements, this work aims in the preparation of α-Ag_2_WO_4_ and β-Ag_2_MoO_4_ powders by the coprecipitation method and further immobilization of such semiconductors into the polymeric (PVA) fibers through the electrospinning method to obtain polymeric mats of PVA, PVA/α-Ag_2_WO_4_, and PVA/β-Ag_2_MoO_4_. The intent is to carry out an investigation of the antimicrobial properties of the α-Ag_2_WO_4_ and β-Ag_2_MoO_4_ powders and compare them with respective PVA/α-Ag_2_WO_4_ and PVA/β-Ag_2_MoO_4_ mats. The hypothesis to be evaluated is that such materials can inhibit the growth of Gram-negative and Gram-positive bacteria.

## 2 Experimental Procedure

### 2.1 Materials

Poly(vinyl alcohol) (PVA) powder with *M̅*_w_ ranging from 89,000 to 98,000 g mol^–1^, degree of hydrolysis (DH) >99%, and a density of 1.3 g cm^–3^ was supplied by Neon, Brazil; sodium tungstate (Na_2_WO_4_·2H_2_O, 99.9%), sodium molybdate (Na_2_MoO_4_·2H_2_O, 99.9%), and silver nitrate (AgNO_3_, 99.9%) were supplied by Sigma-Aldrich.

### 2.2 Preparation of Ag_2_WO_4_ and Ag_2_MoO_4_ Powders

Powders constituted by micro- and nanocrystals of silver tungstate (α-Ag_2_WO_4_) and silver molybdite (β-Ag_2_MoO_4_) were obtained by the coprecipitation method at room temperature (close to 25 °C). Initially, separated solutions of the lattice former salt (2.5 × 10^–3^ mol of Na_2_WO_4_·2H_2_O or Na_2_MoO_4_·2H_2_O) and of AgNO_3_ (5.0 × 10^–3^ mol) were prepared by dissolving the solid on 45 mL of deionized water separately using 50 mL Falcon tubes. Then, the solution containing the Ag^+^ ions was slowly added to the solution containing the WO_4_^2–^ or MoO_4_^2–^ ions and kept under constant stirring for 20 min. The formed solid (white crystals) was washed several times to remove the remaining sodium ions and nitrates. The resulting material was collected, dried at 70 °C for 12 h, and then stored in powder form.

### 2.3 Preparation of Poly(vinyl alcohol) Nanofibers (FPVA), PVA/α-Ag_2_WO_4_ (FPAW), and PVA/β-Ag_2_MoO_4_ (FPAM) Polymeric Mats

**Figure 1:**
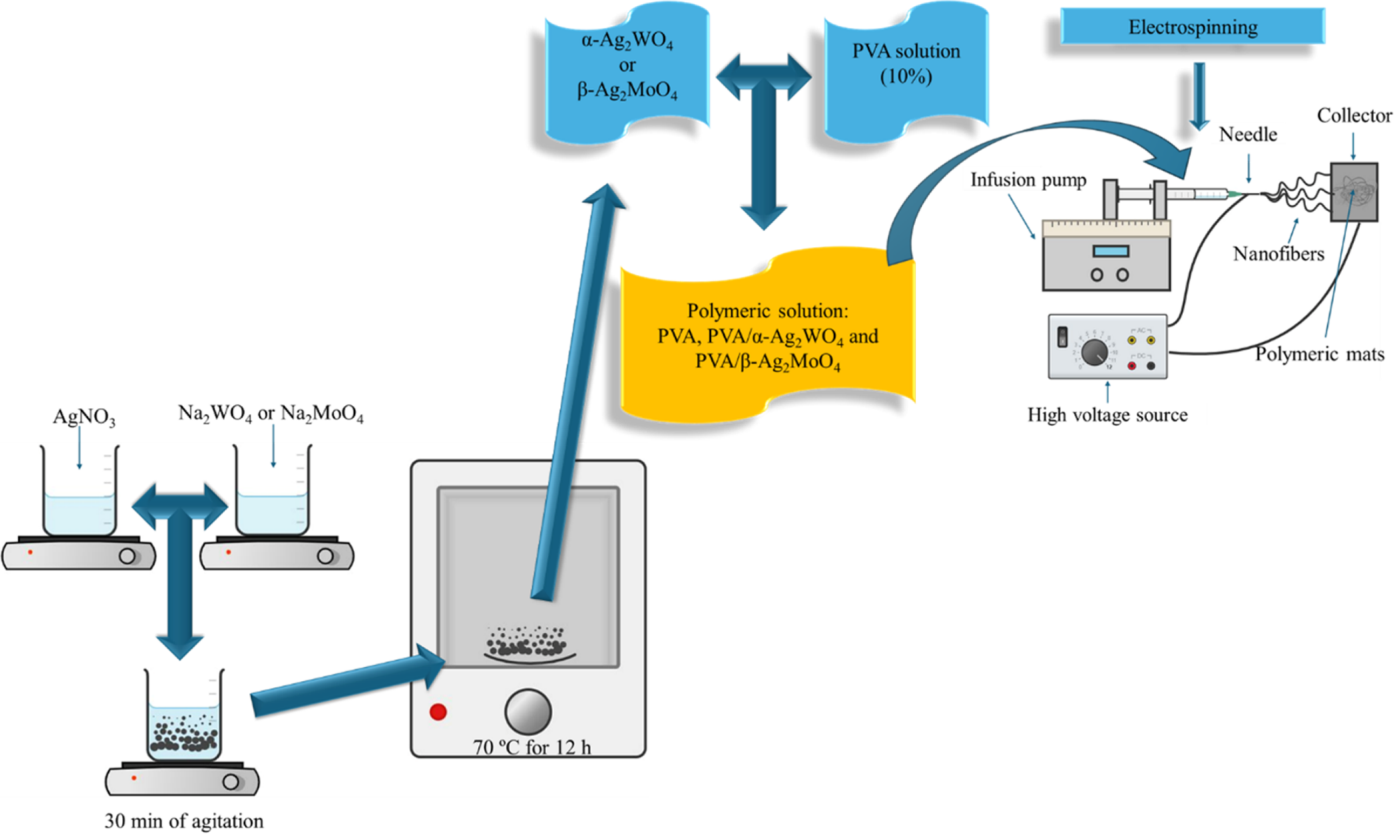
Scheme used for preparing the PAW and PAM powders and FPVA, FPAW, and FPAM polymeric mats.

Initially, a 10% (m/v) aqueous PVA solution was prepared. To prepare the FPAW and FPAM mats, 0.5 g of powder (silver tungstate or silver molybdate) was added into 5 mL of aqueous PVA solution, and the mixture was stirred for 1 h at room temperature. The mixture was ultrasonicated (MYLABOR, SSBu-Batch) for 30 min to disperse the inorganic materials. After complete dispersion, the mixture was placed in a syringe with a 0.8 mm internal diameter capillary needle. This suspension was electrospun with the working parameters for electrospinning fixed as (i) 15 kV for electrical voltage, (ii) 0.5 mL h^–1^ for flowing rate, and (iii) 15 cm as the distance from the needle to the metal collector. The relative humidity of the air was measured and was in the range of 48–55%, and the ambient temperature was controlled at about 25 °C. To prepare the PVA mats (used as a control), 5 mL of the aqueous PVA solution (10% w/v) was placed in a syringe 0.8 mm in diameter. For the electrospinning procedure, the parameters used were the same as those previously mentioned. The samples were labeled as the following: PAW (for α-Ag_2_WO_4_ powder); PAM (for β-Ag_2_MoO_4_ powder); FPVA, FPAW, and FPAM (for PVA, PVA 10%/α-Ag_2_WO_4_, and PVA 10%/β-Ag_2_MoO_4_ mats, respectively). The procedures for the preparation of PAW, PAM, FPVA, FPAW, and FPAM are shown in the scheme in Figure 1.

### 2.4 Characterization

The following techniques were used for the characterization of powders and nanofibers: X-ray diffraction (XRD) was performed using a Shimadzu diffractometer model LABX—XRD 6000, Cu Kα (λ = 1.5406 Å), a range of 2θ from 5 to 80°, with a scan rate of 2° min^–1^. Fourier transform infrared spectra (FTIR) were collected using a Varian instrument model 660-IR spectrometer (in the range of 400–4000 cm^–1^); Raman spectra were acquired in a Senterra spectrometer (Bruker, Germany) equipped with a He–Ne laser (λ = 785 nm) and charge coupled device (CCD) operating from 50 to 950 cm^–1^. Thermal gravimetric analysis (TGA) was performed using an SDT Q600 V20.9 Build 20 TA Instruments. The required mass of the sample (5 ± 0.5 mg) was placed in an alumina pan and heated in a temperature range of 25–700 °C, in a gradient 10 °C min^–1^ and under argon flux 50 mL min^–1^. The optical properties were investigated by a UV–vis spectrophotometer using a Shimadzu UV-3600 diffuse reflectance spectrometer (DRS). Scanning electron microscopy (SEM) images were acquired on a scanning electron microscope with field emission gun (FEG), FEI brand, model Quanta FEG 250, at an acceleration voltage ranging from 1 to 30 kV, equipped with energy dispersive X-ray spectroscopy silicon drift detectors (SDD), Bruker brand, model Quantax EDS, detector XFlash 5010.

### 2.5 Evaluation of Cellular Cytotoxicity

#### 2.5.1 Maintenance of Adherent Cells

Renal epithelial cells of the VERO strain (Banco de Células do Rio de Janeiro, Rio de Janeiro, Brazil) were cultured in Dulbecco’s modified Eagle’s medium (DMEM) and supplemented with 2 mM of l-glutamine, 1.0 g L^–1^ of glucose and 10% of simulated body fluid (SFB), 5000 U mL^–1^ of penicillin, and 5 mg mL^–1^ of streptomycin at 37 °C in an oven with an atmosphere of 95% air and 5% CO_2_. The cell culture was observed daily under an inverted microscope, and the medium was changed when an acidic pH < 7 was observed. After the formation of the cell monolayer, to promote the continuous maintenance of the cells of this lineage, the adhered cells were removed with the enzymatic aid of trypsin (0.25%, sterilized by filtration, 2.5 g of porcine trypsin, 0.2 g of ethylenediaminetetraacetic acid (EDTA), 4Na per litter of Hanks’ balanced salt solution with phenol red) resuspended in DMEM medium and 10% SFB (inactivated at 56 °C, pH 7.4).

#### 2.5.2 Cytotoxicity Test on Renal Epithelial Cells of the Vero Strain

The assays of cytotoxicity with renal epithelial cells (VERO strain) were performed with the reactant 3-(4,5-dimethylthiazol-2-yl)-2,5-diphenyltetrazolium bromide (MTT assay) tetrazolium salt by succinate dehydrogenase enzymes in the mitochondria of cells with the production of insoluble formazan crystals. A suspension of epithelial cells of the VERO lineage (density 2.5 × 10^5^ cell mL^–1^) in DMEM supplemented with 10% of inactivated SFB was dispensed in a sterile plate of 24 cavities and incubated for 24 h at 37 °C and a tension of 5% of CO_2_. After this period, the supernatant was removed. Sections of the materials were added in increasing concentrations (0.25–1000 μg mL^–1^) while the materials were arranged in the wells with sterile tweezers. Then, the cells were incubated for another 48 h under the same conditions. After the incubation period, the cells were washed with 0.01 mol L^–1^ saline-phosphate buffer (PBS), and 300 μL of MTT (2.0 mg mL^–1^) was added in the plate wells, followed by the incubation at 37 °C for 4 h under a light shield. After this period, 300 μL of dimethyl sulfoxide (DMSO) was added to solubilize the purple crystals. The reading was performed through the microplate reader (Power Wave XS, Microplate Reader (BIOTEK)) at 570 nm.^29^ The absorbance values were plotted as a function of the concentration to obtain the CC_50_ value (cytotoxic concentration of 50%) calculated by a simple linear regression.

### 2.6 Antimicrobial Activities

The antimicrobial tests were performed according to the methodology described by Berton et al. (2020). The antimicrobial activities of polymeric mats and powders against *Pseudomonas aeruginosa* (ATCC 27853) and *Staphylococcus aureus* (ATCC 25923) were investigated by the minimum inhibitory concentration (MIC) and minimum bactericidal concentration (MBC). The PAW and PAM (2 mg mL^–1^) were dispersed in sterilized distillates using an ultrasonic apparatus at 42 kHz for 25 min (25 °C), while the FPVA, FPAW, and FPAM mats were in the form of disks with a diameter of 6.0 mm, having been sterilized under ultraviolet (UV) exposure for 10 min. The tests were performed using the microdilution of Mueller-Hinton broth at pH 7.4 ± 0.2 (37 °C for 24 h) in the concentration range of 62.5–0.48 mg mL^–1^. The microbial density in an aqueous solution of NaCl (0.85 wt %) was 1.0 × 10^8^ colony-forming units (CFU mL^–1^). MIC is defined as the lowest concentration that prevents the visible growth of microbial cells. MBC is the lowest concentration of a material that can kill microbial cells. MBC was determined using a 10 μL cell culture without visual bacterial growths on Mueller-Hinton agar at pH 7.4 ± 0.2, 37 °C for 24 h.^29^ The digital images obtained from the MIC and MBC procedures are presented in Tables S1 and S2 of the Supporting Information.

## 3 Results and Discussion

### 3.1 XRD Analysis

**Figure 2:**
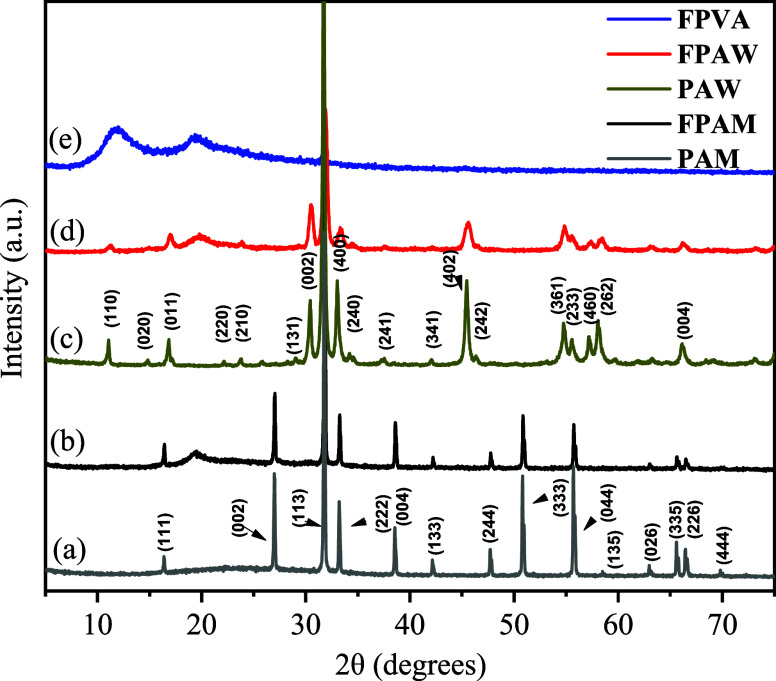
XRD patterns of the samples: (a) PAM, (b) FPAM, (c) PAW, (d) FPAW, and (e) FPVA.

The samples were characterized by X-ray diffraction (XRD) to evaluate long-range structural order and disorder. The XRD patterns of PAW and PAM powders and FPVA, FPAW, and FPAM polymeric mats are presented in Figure 2.

According to the analysis of the XRD profile illustrated in Figure 2a, all diffraction peaks can be accurately indexed to the cubic structure of β-Ag_2_MoO_4_. The positions of all diffraction peaks match the pattern defined in the Inorganic Crystal Structure Data (ICSD) standard card # 36187.^21^ The diffraction peaks of PVA and β-Ag_2_MoO_4_ are seen in the XRD profiles of the FPAM mats in Figure 2b. A decrease in the diffraction peaks’ intensity compared to the separated components (Figure 2a,e) can be observed. The peak at 2θ = 16.5° identified in the XRD pattern of FPAM can also be observed in the profile of FPVA. As the other peaks of PAM are also observed in the XRD profile of FPAM, the XRD profile of FPAM indicates that the FPAM fibers are composed of PVA and β-Ag_2_MoO_4_.

Figure 2c exhibits the diffraction peaks characteristic of α-Ag_2_WO_4_. All peaks can be exactly indexed to the single-phase orthorhombic structure, matching the peaks defined in the ICSD standard card # 4165.^30^ A decrease in the intensities of the diffraction peaks for the FPAW system relative to PAW can be seen in the XRD profile (Figure 2d). The peak at 2θ = 16.5° identified in the XRD pattern of FPAW can be observed in the XRD profile of FPVA. As the other peaks of PAW are also observed in the XRD profile of FPAW, the XRD profile of FPAW indicates that the FPAW fibers are composed of PVA and α-Ag_2_WO_4_.

The XRD pattern of PVA in Figure 2e presents two peaks, one at 7.5° and the other at 16.5°. The degree of crystallinity of PVA depends on its molecular structure, including stereospecificity, the 1,2-glycol group, branches, and acetyl groups in the 30 chains.^31,32^ Under normal conditions, polymers do not crystallize entirely due to the size of their chains, resulting in the formation of ordered and disordered regions influenced by several factors, the cooling rate among them.^27,33^ According to the results, there was a reduction in the intensity of the diffraction peaks of PVA on both XRD profiles (FPAM and FPAW mats) compared with the XRD profile of PVA fibers. The peak at 7.5° can be attributed to the formation of parallel planes due to the annealing of PVA, and the peak at 16.5° can be explained by the semicrystalline orthorhombic structure of PVA.^33^

### 3.2 Analysis through FTIR Spectroscopy

**Figure 3:**
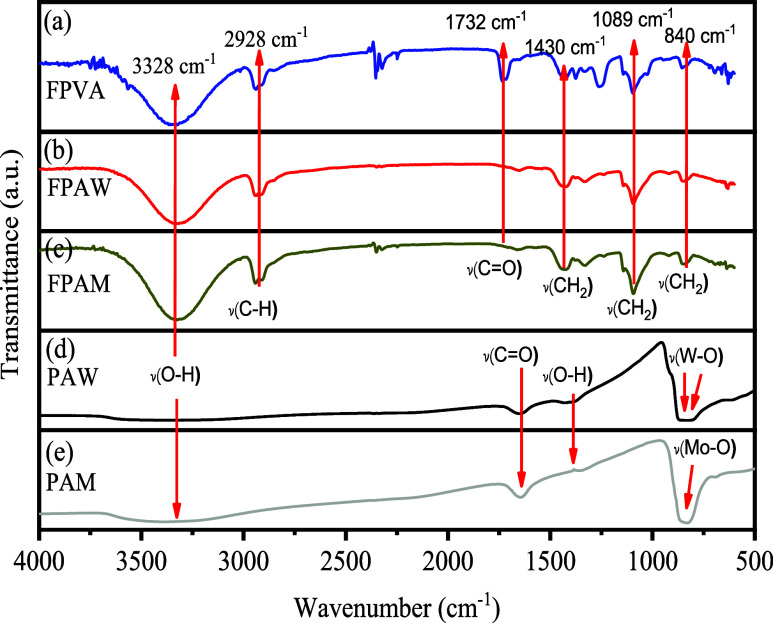
FTIR of the samples: (a) FPVA, (b) FPAW, (c) FPAM, (d) PAW, and (e) PAM.

FTIR spectroscopy was used to elucidate the chemical groups present in the FPVA, FPAW, and FPAM polymeric mats, as well as PAW and PAM powders. FTIR spectra of FPVA, FPAM, FPAW, PAW, and PAM are presented in Figure 3. Characteristic bands of the chemical groups present on PVA, α-Ag_2_WO_4_, and Ag_2_MoO_4_ were observed on the FPVA, FPAW, and FPAM spectra, respectively. In the spectra shown in Figure 3a–c for FPVA, FPAW, and FPAM fibers, the following bands were observed: 3330 cm^–1^, attributed to the O–H stretching; 2929 cm^–1^, attributed to the C–H of the alkyl groups; 1720 cm^–1^, corresponding to C=O of the carbonyl groups; 1430 cm^–1^, attributed to stretching modes of alcohol groups, asymmetric stretching bands CH_2_ of the methyl group at 1089 cm^–1^ (bending) and 840 cm^–1^ (twisting), respectively.^27^ Two intense absorption bands at 828 and 862 cm^–1^ were observed in the FTIR spectrum of Figure 3d for PAW. These modes are ascribed to O–W bonds and antisymmetric stretching within distorted [WO_6_] clusters.^7^ Only one intense absorption band at 838 cm^–1^ was observed in the PAM spectrum (Figure 3e). This was ascribed to bonds between O–M and stretching vibrations within distorted [MoO_4_] clusters.^21^

### 3.3 Raman Spectroscopy Analysis

**Figure 4:**
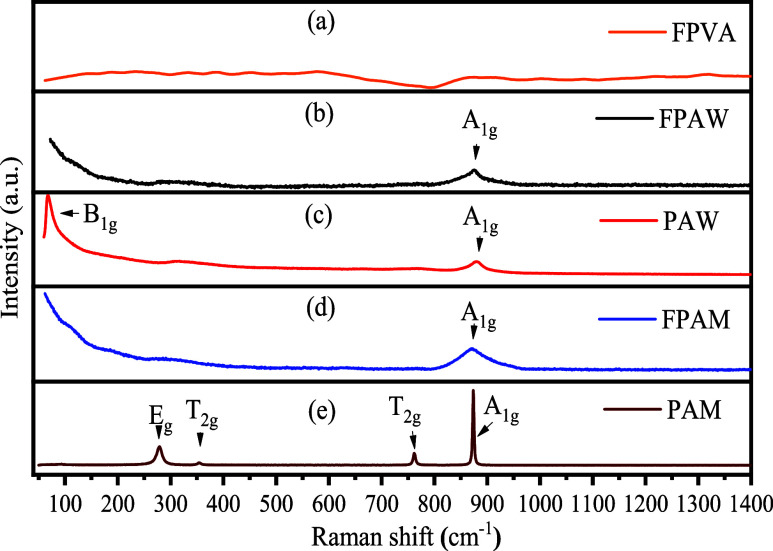
Raman spectra of the samples: (a) FPVA, (b) FPAW, (c) PAW, (d) FPAM, and (e) PAM.

Figure 4 shows the Raman spectra of FPVA, FPAW, and FPAM mats, as well as of PAM and PAW powders. No active modes were verified on the Raman spectra of FPVA (Figure 4a). Besides only a very broad band of low intensity, the Raman spectra for FPAW and FPAM samples (Figure 4b,d) exhibited active modes corresponding to the PAW and PAM powders, around 880 cm^–1^, albeit with low intensity. Figure 4c shows the Raman spectrum of the PAW sample. For this, 18 to 21 active vibrational modes are expected in Raman spectroscopy; however, some modes are not detected due to their low intensities.^11,34^ This spectrum shows a high degree of short-range structural order in the lattice. The most intense A_1g_ band observed around 880 cm^–1^ originates from the symmetric stretching vibration of a short terminal W–O bond. Other bands are contributed by asymmetric stretching vibration modes of longer W–O bonds, symmetric stretching vibrations of longer W–O bonds, deformation vibrations of short W–O bonds, interchain deformation modes, and lattice modes of [WO_4_] clusters.^11,34^

Figure 4e displays the Raman spectrum of the PAM sample. Four Raman-active bands were identified in this spectrum, located between 50 and 1000 cm^–1^. All these bands are intense and well-defined, suggesting that all PAM microcrystals are structurally ordered at short range.^7^ The E_g_ band situated at 277 cm^–1^ is attributed to the external structural vibrations of the octahedral [AgO_6_] clusters. At the same time, the T_2g_ modes found at 353 and 761 cm^–1^ are related to torsional vibrations of oxygen and molybdenum atoms in the O–Mo–O bonds within the tetrahedral [MoO_4_] clusters. The A_1g_ band at 880 cm^–1^ is ascribed to symmetric stretching vibrations of the oxygen atoms in the [MoO_4_] clusters.^7,21^ As expected, no modes related to PVA were observed in the FPAW and FPAM spectra. So, it is possible to differentiate the FPVA sample and FPAW (or FPAM) using Raman spectroscopy due to the presence of specific Raman modes of the α-Ag_2_WO_4_ (or β-Ag_2_MoO_4_).

### 3.4 TGA Analysis

**Figure 5:**
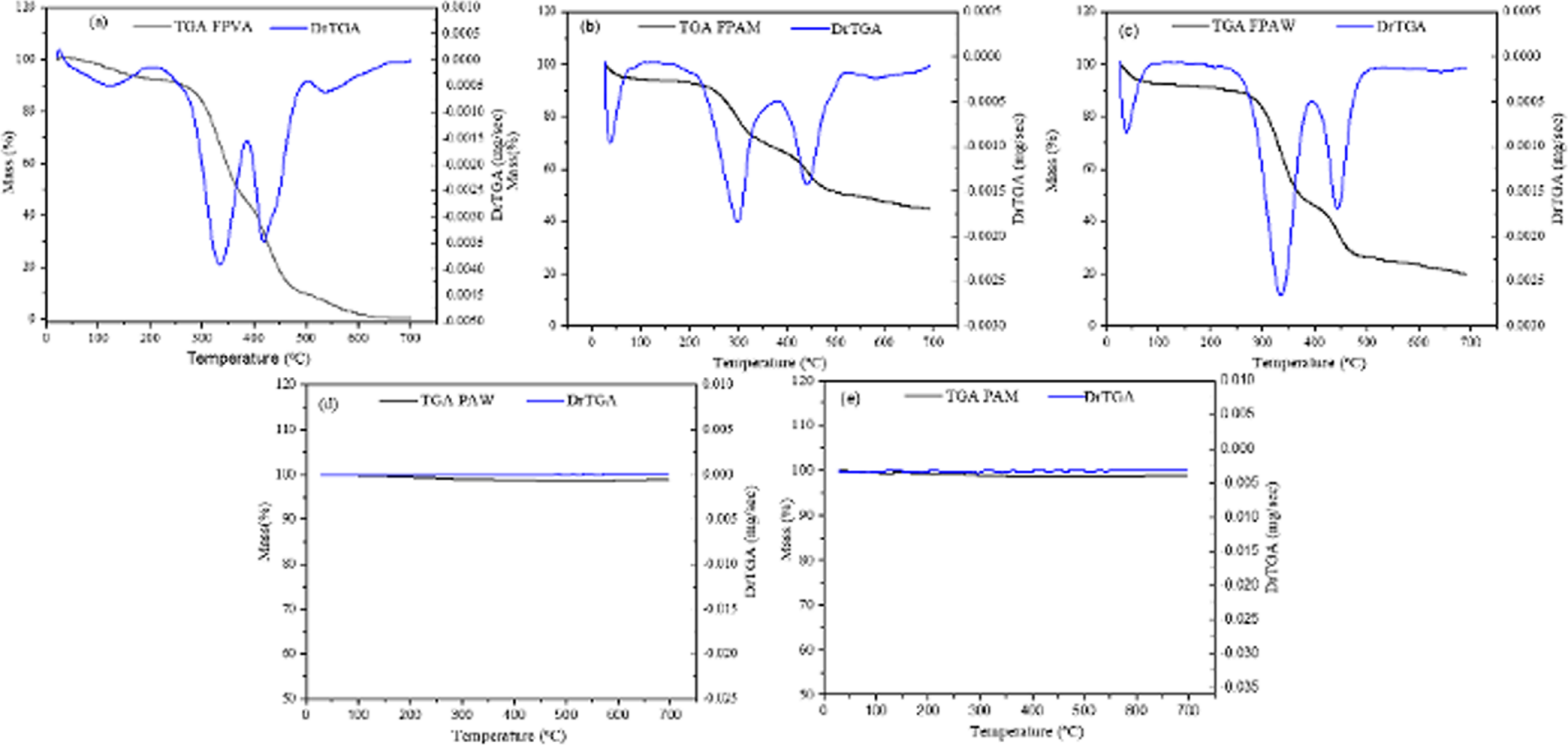
TGA and DrTGA curves for (a) FPVA, (b) FPAM, (c) FPAW, (d) PAW, and (e) PAM.

Figure 5a–e shows the TG curves of FPVA, FPAW, and FPAM mats and PAW and PAM powders. As can be seen in Figure 5a, the FPVA sample completely decomposed at 450 °C. It was found in the literature that PVA degrades in the range from 300 to 400 °C.^32,33^ On the other hand, the thermal decomposition of the FPAW and FPAM polymeric mats occurred in three stages (loss of moisture, decomposition of organic matter, and stability of the formed material), reaching a total mass loss of approximately 80 and 60%, respectively, at 700 °C. The PAW and PAM samples showed practically no loss of mass. In this case, both thermogravimetric curves demonstrate good thermal stability until 700 °C.

### 3.5 Scanning Electron Microscopy (SEM) Analysis

**Figure 6:**
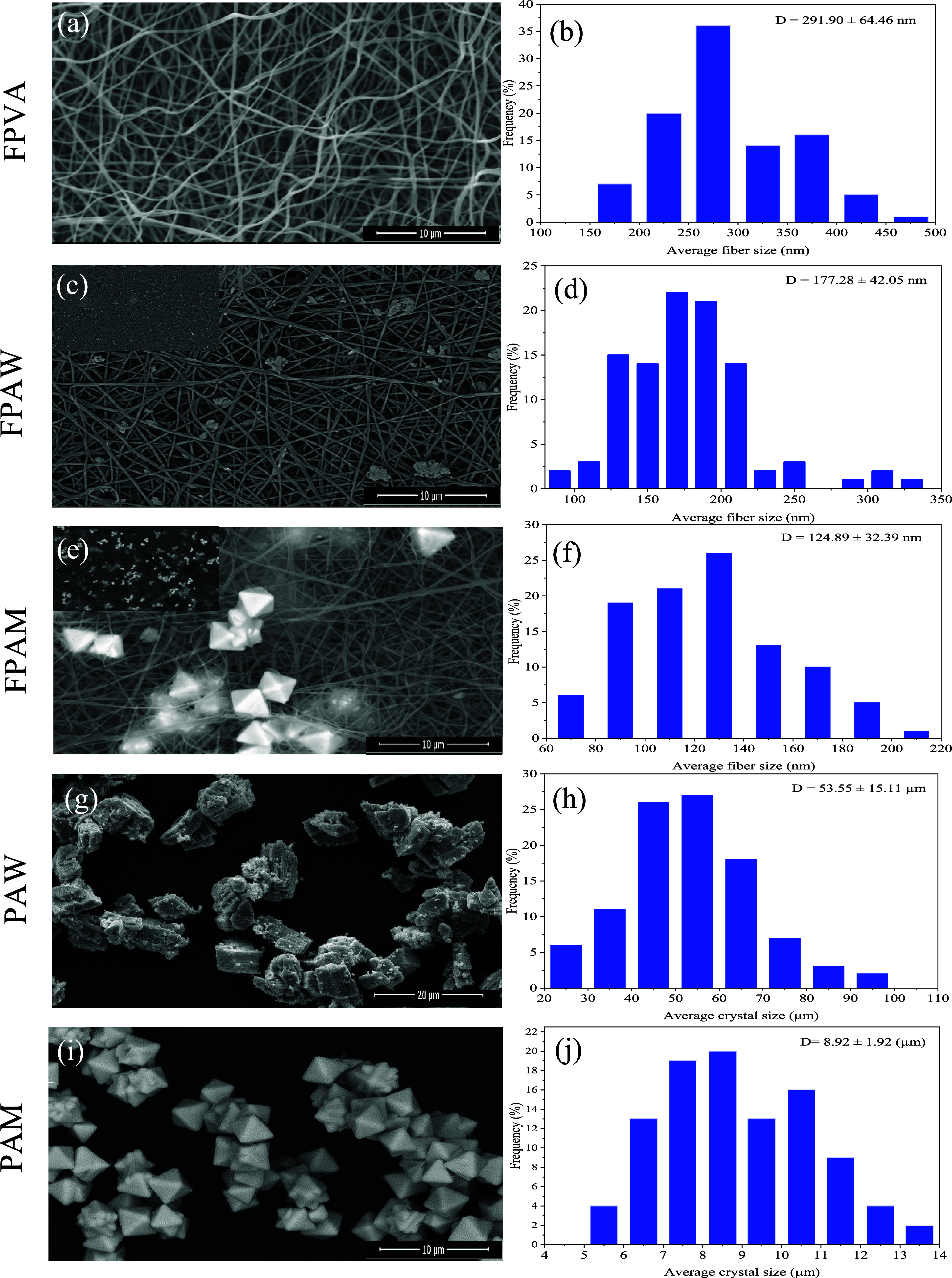
SEM (a, c, e, g, i), fiber, and particle (b, d, f, h, j) diameters in the FPVA polymeric mats (a, b), FPAW polymeric mats (c, d), FPAM polymeric mats (e, f), PAW powder (g, h), and PAM powder (i, j).

Figure 6 shows the acquired SEM images from FPVA, FPAW, and FPAM polymeric mats and PAW and PAM powders. Figure 6a shows an SEM image of the FPVA electrospun fibers. As shown in Figure 6b, FPVA fibers do not present uniformity in fiber diameters, presenting in fact a wide distribution of diameters of about 291.90 ± 64.46 nm. The same behavior of nonuniformity of fiber diameters can be observed on SEM images of Figure 6c and 6e, for the FPAW and FPMA samples, respectively, with an average diameter of 177.28 ± 42.05 and 124.89 ± 32.39 nm according to Figure 6d and 6f. With this, it can be observed that the presence of the semiconductor α-Ag_2_WO_4_ or β-Ag_2_MoO_4_ in the fibers based on PVA caused a decrease in the fibers’ average diameter. The electrical conductivity of a polymeric solution is determined by a sum of factors that include the type/structure of the polymer, the eventual presence of salts in the solution, and the solvent used.^22,35,36^ When a high voltage is applied during the electrospinning process, the charges are transferred to the polymer solution and, allied to the conductivity of the solvent, in general, lead to an increase in conductivity and a decrease in the average diameter of the fibers.^22,35^Figure 6g and 6i indicate that PAW and PAM powders have nonuniform and agglomerated particles with average diameters of 53.55 ± 15.11 and 8.92 ± 1.92 μm, respectively, as can be seen in Figure 6h and 6j. The EDS spectra of the FPVA, FPAW, FPAM, PAW, and PAM samples are illustrated in Figures S1–S5 in the Supporting Information.

### 3.6 UV–Vis Absorption Spectroscopy Analysis

**Figure 7:**
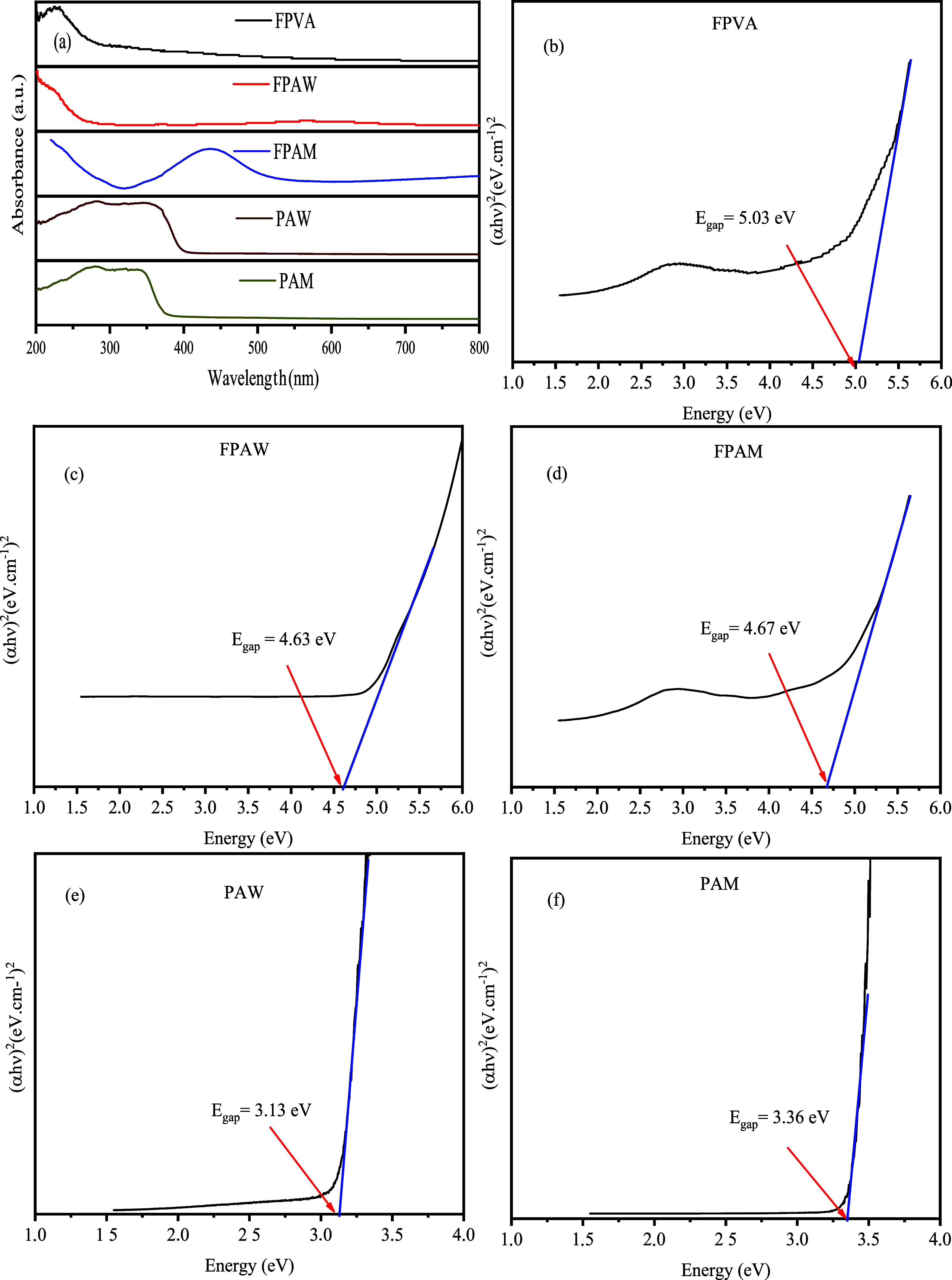
(a) UV–visible absorption spectra and Tauc plots of (b) FPVA, (c) FPAW, (d) FPAM, (e) PAW, and (f) PAM samples.

Figure 7 shows the UV–vis absorption spectra and the values of the optical band gap obtained for the composite polymeric mats (FPVA and FPAW) and PAW and PAM semiconductors.

According to the literature,^7,11^ tungstate and molybdate each have one typical optical absorption process characterized by direct electronic transitions, which occur from maximum energy states (located near or in the valence band) to minimum energy states (located below or in the conduction band) in the same regions in the Brillouin zone. Considering this information, the *E*_gap_ values of the polymeric mats and semiconductors, FPVA, FPAW, FPAM, PAW, and PAM, were calculated by extrapolating the linear portion of the Tauc plot curves.^7,11^ The results obtained are illustrated in Figure 7b–f.

The UV absorptions observed in the ultraviolet–visible spectrum can be attributed to the transitions of the polymer’s highest-occupied molecular orbital (HOMO) to lowest-unoccupied molecular orbital (LUMO) orbitals and transitions from the valence bands to the conduction bands in silver-based semiconductors.^33^Figure 6a shows that the polymer matrix presents an increase in the spectrum of absorbance around 257 nm, which is associated with the π → π* transitions of the groups present in the FPVA.^7^ In addition, silver-based semiconductors exhibit an absorption around 380 nm.

The band gap energies (*E*_g_) were experimentally estimated by extrapolating the linear portion of the curves of the Tauc graph, as shown in Figure 7b–f. Although pure PAW and PAM have lower *E*_g_ (3.13 and 3.36 eV, respectively) than the composite fiber’s HOMO to LUMO transition, FPAW (*E*_g_ = 4.63 eV), and FPAM (*E*_g_ = 4.67 eV), polymer mats have *E*_g_ values close to the transition observed for pure FPVA (*E*_g_ = 5.03 eV). This may be associated with the indirect transition behavior of the excitation mechanism of these metal oxides, which could bring a superposition of the interband transition of the metal oxide with the HOMO to LUMO transition of the polymer.^7^ Thus, the curve of the Tauc graph shows only one transition.^33,37^

### 3.7 Evaluation of Cytotoxicity

The *in vitro* cytotoxicity study aims to evaluate the presence or absence of toxicity of a substance or compound in living cells. Usually, this is done using cell cultures, and it is used to define and provide meaningful information about parameters such as genotoxicity, mutation induction, or programmed cell death. By establishing the dose at which 50% (CC_50_) of cells are affected, it is possible to quantitatively compare the responses of individual compounds in different systems or multiple compounds in individual systems.^38^

The evaluation of the cytotoxicity of FPVA, FPAW, and FPAM polymeric mats and PAW and PAM powders against VERO cells was performed to assess the cell viability through the MTT assay. Vero cells were maintained for 48 h in the presence or absence of the materials, and the toxic effect (Table 2) was expressed through the CC_50_ using the MTT method compared to the positive cytotoxicity control, doxorubicin.

The literature describes several types of cytotoxicity assays. Among them, we highlight the MTT used in our study. In the MTT assay, the tetrazolium salt [3-(4,5-dimethylthiazole-2-yl)2,5-diphenyltetrazolium bromide], a water-soluble compound, which has a pale yellow color in aqueous solution, is easily incorporated by viable cells, which then reduce this compound in their mitochondria by dehydrogenases.^39^ Once reduced, MTT is converted to a dark blue formazan compound stored in the cell cytoplasm.^40^ After adding an appropriate solvent, the product is solubilized, released, and spectroscopically quantified.^40^ The cells’ ability to reduce MTT indicates mitochondrial activity and integrity, which are interpreted as measures of cell viability.^29,39,40^

As expected, the polymeric mats FPVA did not show any toxicity profiles even at the highest concentration tested (1000 μg mL^–1^), which is consistent with data from the literature.^41,42^ In the case of VERO cells, PVA can be considered less toxic due to its inert nature, water solubility, and ability to form biocompatible films.^43^ This indicates that it can be classified as more environmentally friendly, especially compared to other synthetic polymers that are more toxic or persistent in the environment.^43^ However, despite its low toxicity to eukaryotic cells like VERO cells, PVA can inhibit the growth of bacteria of different types.^43^ This inhibition may occur due to PVA’s ability to form a physical barrier when in contact with bacterial cells, which could interfere with and make difficult the nutrients transport through the bacterium cell wall, thus actuating metabolic processes essential for bacterial growth and proliferation.^43^

Meanwhile, the treatment with mats impregnated with metal oxides, specifically silver molybdate and silver tungstate, exhibited some level of toxicity, although it was not significant when compared to the toxic profile observed with doxorubicin. Approximately 1 mg of the FPAW and FPAM materials was able to inhibit cell growth by 87 and 73%, respectively. These data correlate with the CC_50_ values found (Table 1) for the same materials, 103.70 ± 18 and 111.22 ± 4 μg mL^–1^. An important point to highlight is the toxicity profile of the isolated metal oxides PAW and PAM, whose inhibition rate in Vero cells was 21.74 and <15 μg mL^–1^, respectively.

**Table 1:** Results (CC_50_) from Cytotoxicity Assays Using Polymeric Mats (FPVA, FPAW, and FPA), as well as PAW and PAM against Vero Cells

materials	average (μg mL^–1^)
FPVA	>1.000
FPAW	103.70 ± 18.90
FPAM	111.22 ± 4.02
PAW	21.74 ± 0.04
PAM	<15
adoxorubicin	0.01 ± 0.0^b^

a Doxorubicin, positive control for cytotoxicity.

b Concentration in micromolar (μM).

### 3.8 Antimicrobial Performance

To confirm the microbiological activities of the FPVA, FPAW, and FPAM mats and PAW and PAM powders, the antimicrobial assays against Gram-negative bacteria, *P. aeruginosa* (ATCC 27853), and Gram-positive *S. aureus* (ATCC 25923) were performed and compared, after 24 h of exposure, in the concentration range of 62.5–0.48 mg mL^–1^. The MIC/MBC results are listed in Table 2. Based on such a table, it can be verified that all samples exhibited antimicrobial activity for the minimum inhibitory concentration (MIC) against both types of bacteria, *P. aeruginosa* and *S. aureus*, and the lowest values were for the PAW sample (MIC = 0.014 mg mL^–1^). The MBC for the FPAW and FPAM samples was equal to 1.35 and 3.67 mg mL^–1^, respectively, being lethal against *S. aureus*. The PAM, FPAW, and FPAM samples showed MBC values (mg mL^–1^) equal to 1.43, 1.35, and 11.03 for the bacterium *P. aeruginosa*. FPVA and PAW did not present MBC for both types of bacteria.

**Table 2:** Minimum Inhibitory Concentration (MIC) and Minimum Bactericidal Concentration (MBC) Evaluated for the Samples (pH 7.4 ± 0.2) at 37 °C after 24 h^a^

	MIC/MBC (mg mL^–1^)				
bacterium	FPVA	FPAW	FPAM	PAW	PAM
*P. aeruginosa* ATCC 27853(N)	8.78/ND	1.35/1.35	3.68/11.03	0.014/ND	1.43/1.43
*S. aureus* ATCC 25923(P)	14.63/ND	1.35/1.35	3.68/3.67	0.794/ND	1.43/ND

a ND-not detected.

Li et al. studied the development and evaluation of ZnIn_2_S_4_/Ag_2_MoO_4_ (ZZA) composite photocatalytic nanofibers using a Z-scheme for antibacterial and photocatalytic applications under visible light.^44^ The composite demonstrated enhanced photocatalytic efficiency in the degradation of organic pollutants under visible light compared to the individual components (ZnIn_2_S_4_ and Ag_2_MoO_4_).^44^ Additionally, the composite was tested against *S. aureus* and *P. aeruginosa* to assess its antibacterial efficacy.^44^ The results showed that the ZZA-20 composite exhibited the highest antibacterial efficacy, with the lowest MIC values of 10 mg mL^–1^ and MBC values of 20 mg mL^–1^ for both tested bacteria, followed by ZZA-10 and ZZA-5.^44^ Bastos and collaborators investigated the performance of silver tungstate microcrystals against various multidrug-resistant clinical microorganisms.^12^ One of the points addressed was the minimum inhibitory concentration (MIC) of silver tungstate microcrystals for the bacterium *P. aeruginosa*. According to these authors, the MIC value for *P. aeruginosa* was 62.5 μg mL^–1^.^12^^12^

The antimicrobial action of metal semiconductors is associated with four mechanisms: (i) adhesion of semiconductors to the membrane of the cell wall; (ii) penetration of semiconductors into cells causing damage to intracellular structures (mitochondria, vacuoles, ribosomes) and biomolecules (proteins, lipids and DNA); (iii) cellular toxicity and oxidative stress caused by reactive oxygen species (ROS) and free radicals; and (iv) modulation of signal transduction pathways.^5,45^ Even so, modulation of the immune system of cells can occur, orchestrating the inflammatory response, which assists in inhibiting microorganisms.^5^

In this context, the literature reports that exposing silver-based semiconductors to microorganisms causes their adhesion to the cell wall and membrane. So, the positive surface charge of the antimicrobial agent is crucial for adhesion.^5,45^ Thus, the electrostatic attraction between the semiconductor and the negatively charged cell membrane of the microorganism facilitates fixation on cell membranes. This morphological change becomes evident after such interactions and can be characterized by shrinkage of the cytoplasm, the detachment of the membrane, and the ultimate rupture of the cell wall.^46^

The greater effectiveness of antimicrobial therapy on Gram-negative bacteria than on Gram-positive bacteria is due to the cell wall of the latter being thicker.^46^ In Gram-positive bacteria, the thicker cell wall, as well as the negative charge of the peptidoglycan, causes Ag^+^ ions to become trapped in the cell wall, resulting in fewer antimicrobial agents within the cell. This contrasts with Gram-negative bacteria with a thinner cell wall and less peptidoglycan.^34,46^

The samples PAW and PAM exhibit a more positive zeta potential compared to the FPVA, FPAW, and FPAM mats (see Figure S6 in the Supporting Information). Consequently, a lower MIC value would be expected for *P. aeruginosa* (Gram-negative) compared to *S. aureus* (Gram-positive), which was observed only for the PAW sample. The other samples exhibited equal MIC values for both types of bacteria. Another relevant point to highlight is that the FPAW and FPAM mats (slightly positively charged) demonstrated MIC and MBC for both bacteria, thus showing their high efficacy.

Inhibition is associated with the morphological and physiological characteristics of Gram-negative bacteria.^6,30^ The destruction of the cell wall by microcrystals (PAW and PAM) and mats (FPAM and FPAW) occurs through physical interaction.^6,30^ Another possible factor contributing to the vulnerability of Gram-negative bacteria to PAW and PAM microcrystals may be the presence of negatively charged lipopolysaccharides.^6,30^ The production of reactive oxygen species (ROS) by microcrystals, whose surfaces exhibit a positive charge due to angular clusters [AgO_2_] and heterojunctions, results in a higher affinity for negatively charged molecules.^30^ This promotes the interaction between bacteria and the high-electron-density surface of the microcrystals, favoring the oxidation and rupture of carbon chains in the bacterial membrane.^30^

The powerful effect of the antimicrobial activity of metal ions is associated with their ability to produce reactive oxygen species (ROS) and free radicals such as the hydroxyl radical (OH^•^), peroxide (H_2_O_2_), superoxide anion (O_2_^–^), singlet oxygen, etc. However, the mechanism is still somewhat complex.^47,48^ The band gap is a key parameter in determining the cytotoxic and bactericidal efficiency of semiconductors, as it regulates the material’s ability to generate reactive oxygen species (ROS) under light irradiation. Materials semiconductors with a wider band gap remain relevant in specific contexts involving UV light.^45−47^ For silver-based semiconductors, the main antimicrobial action is associated with generating ROS and free radicals, which consequently increases the oxidative stress on bacterial cells.^5,45−47^

These studies demonstrated that electrospun polymeric fibers containing silver-based semiconductors offer an alternative tool for antimicrobial action. The fact that the semiconductor is supported on the fibers leads it to present a specific surface area capable of binding to the bacterial cell wall, thus causing oxidative stress to occur in the cells through the generation of ROS. On the other hand, the higher cell viability of composite fibers (FPAM and FPAW) is attractive because it leads to higher safety for humans in real usage of composite fibers as antibacterial agents.

## 4 Conclusions

Infections caused by pathogens are a growing concern for public health. Consequently, developing new antimicrobial agents with a broad spectrum of activity has become essential to address the increasingly diverse and rising threats posed by microorganisms. In this work, α-Ag_2_WO_4_ and β-Ag_2_MoO_4_ powders obtained by the coprecipitation method and composite polymeric mats obtained through the electrospinning method using the oxide powders and PVA were studied, and their effects cytotoxic and antimicrobial properties were investigated.

The XRD, FTIR, and Raman results revealed that the powders based on α-Ag_2_WO_4_ and β-Ag_2_MoO_4_ were incorporated into the polymeric matrix of PVA originating the composite nanofibers. The SEM results showed the formation of electrospun fibers designated as FPVA, FPAW, and FPAM with an average diameter of 291.90, 177.28, and 124.89 nm, respectively, and nonuniform and agglomerated particles designated as PAW, with an average diameter of 53.55 μm; and PAM, with an average diameter of 8.92 μm, were obtained.

The 50% cytotoxic concentrations (CC_50_) of the FPVA, FPAW, and FPAM mats and PAW and PAM powder, obtained through the microscopic evaluation of the cellular morphological changes for the VERO cells, were estimated by regression analysis with concentrations (μg mL^–1^) of 21.74 ± 0.04 for PAW; <15 for PAM; 103.70 ± 18.90 for FPAW; 111.22 ± 4.02 for FPAM; and >1000 for FPVA. This demonstrates that as the semiconductor is supported in the polymer matrix, its cytotoxic effect decreases considerably.

Our studies demonstrated that crystalline particles of α-Ag_2_WO_4_, β-Ag_2_MoO_4_, and electrospun fibers of PVA/α-Ag_2_WO_4_ and PVA/β-Ag_2_MoO_4_ exhibit antibacterial activity against Gram-negative bacteria *P. aeruginosa* and Gram-positive *S. aureus*. However, the composite fibers and powders exhibited a more significant bactericidal effect against both types of bacteria, and this fact can be attributed to the production of reactive oxygen species responsible for inducing high oxidative stress in the bacteria, thus causing cellular death. However, the immobilization of α-Ag_2_WO_4_ and β-Ag_2_MoO_4_ in the PVA matrix, forming the composite fibers, preserves the bacterial activities and leads to lesser cytotoxicity than immobilized powders. Such methodology is interesting because it makes it safer for humans to use composite fibers as antibacterial agents.

## Supporting Information Available

Detailed experimental methods, characterization by EDS analysis, mechanical properties, ζ potential measures, and digital images of the antimicrobial performance (MIC and MBC) (PDF)

## Abbreviations

α-Ag_2_WO_4_: silver tungstate
β-Ag_2_MoO_4_: silver molybdite
CC_50_: 50% cytotoxic concentrations
DMEM: Dulbecco’s modified Eagle medium
FBS: fetal bovine serum
FPVA: poly(vinyl alcohol) mats
FPAW: silver tungstate mats
FPAM: silver molybdite mats
ICSD: inorganic crystal structure data
PAW: silver tungstate powder
PAM: silver molybdite powder
PBS: phosphate buffered saline
PVA: poly(vinyl alcohol)
MBC: minimum bactericidal concentration
MIC: minimum inhibitory concentration
MTT: 3-(4,5-dimethylthiazol-2-yl)-2,5-diphenyltetrazolium bromide
ROS: reactive oxygen species
