# Supplementary material for: Nanofiber Composites of Poly(vinyl alcohol)/Silver-Based Molybdate and Tungstate Oxide Semiconductors for Antimicrobial Applications
Source: ACS Omega. 2025 Jan 15;10(3):2586–97. doi: 10.1021/acsomega.4c07471 (PMC11780561; doi:10.1021/acsomega.4c07471)
Supplement: Supplementary file 1 — ao4c07471_si_001.pdf [file ao4c07471_si_001.pdf]

NANOFIBER COMPOSITES OF POLY(VINYL ALCOHOL)/SILVER-BASED MOLYBDATE AND TUNGSTATE OXIDE SEMICONDUCTORS FOR ANTIMICROBIAL APPLICATIONS

*Vicente de Sousa Marques[a], Lee Marx Gomes de Carvalho[a], Débora Aparecida de Almeida[b], Rian Richard Santos de Farias[b], Andressa Dalolio Valente[b], Alessandro Francisco Martins[b,c], Celso Nakamura[b], Edvani Curti Muniz[a,b]**

[a] Department of Chemistry, Federal University of Piauí (UFPI), Teresina, PI 64049-550, Brazil.

[b] Department of Chemistry, State University of Maringá (UEM), Maringá, PR 87020-900, Brazil.

[c] Department of Chemistry, Pittsburg State University (PSU), Pittsburg, KS 66762, USA.

* e-mail: munizec@ufpi.edu.br

SUPPORTING INFORMATION

Supporting Information

1. **Characterization**

The following techniques were used for the characterization of powders and nanofibers: X-ray diffraction (XRD) was performed by Shimadzu model LABX - XRD 6000, radiation-Kα (λ =1.5406 Å), range of 2θ from 5 ° to 80 °, with a scan rate of 2 ° min^{-1}. FTIR and Raman spectra were collected using a Varian equipment, model IR 660, in the range of 400 to 4000 cm^{-1} and Senterra spectrometer (Bruker, Germany), equipped with He-Ne laser (λ = 532 nm) and CCD operating from 50 to 950 cm^{-1} respectively. Thermal gravimetric analysis (TGA-DTG) was realized using a SDT Q600 V20.9 Build 20 (TA instruments) equipment. Samples were properly weighed in alumina pans (mass of 5 ± 0.5 mg) and heated in a temperature range of 25-700 ºC at 10 ºC min^{-1} under 10 ºC min^{-1} argon flux. The optical properties were studied by UV-vis using the UV-3600 Shimadzu UV-3600 Diffuse Reflectance Spectrometer (DRS). The zeta potential was performed with a potential instrument Malvern Zetasize Nano-ZS90. SEM micrographs were performed under the scanning electron microscope with field emission cannon, FEI brand, model Quanta FEG 250, with acceleration voltage from 1 to 30 kV, equipped with EDS SDD (Silicon drift detectors), Bruker brand, model Quantax EDS, detector XFlash 5010.

2. **Mechanical Properties**

The mechanical testing, specifically the tensile strength, of pure FPVA fibers, FPAM, and FPAW was determined using a universal tensile and compression testing machine from HSENSOR at room temperature, ca. 25 ºC). The tensile rate was set at 10 mm min^{-1}, and the sample size for tensile mechanical evaluation was 50 x 0.05 mm² (width x thickness). The results were presented as mean ± standard deviation (n = 3).

3. Results and discussion

3.1. EDS analysis

Figures S1-S5 illustrate the EDS spectra of the FPVA, FPAW, FPAM, PAW and PAM samples respectively. As can be seen in the spectrum of Figure S1, we only have the presence of peaks related to the composition of the FPVA, which are carbon and oxygen. It is also possible to verify a peak around 2.1 eV, which refers to the coating of the sample by gold particles. The same behavior mentioned above can be observed in Figure S2, S3, S4 e S5 for the FPAW, FPAM, PAW and PAM samples.

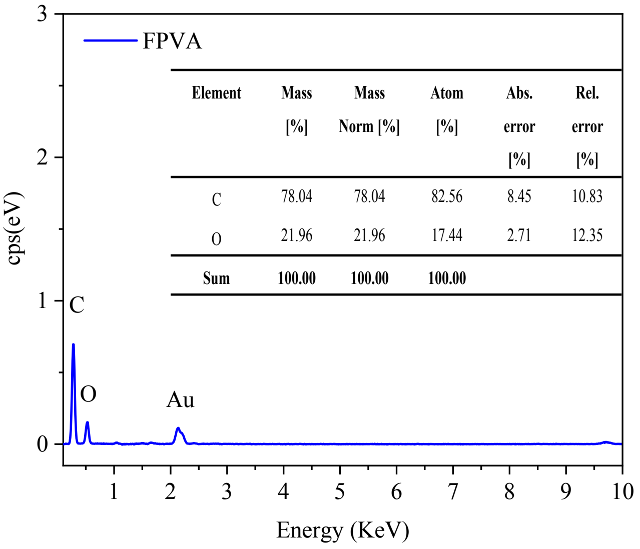

Element	Mass [%]	Mass Norm [%]	Atom [%]	Abs. error [%]	Rel. error [%]
C	78.04	78.04	82.56	8.45	10.83
O	21.96	21.96	17.44	2.71	12.35
**Sum**	**100.00**	**100.00**	**100.00**		

Figure S1. Spectra EDS of the FPVA polymeric mats obtained by the electrospinning method.

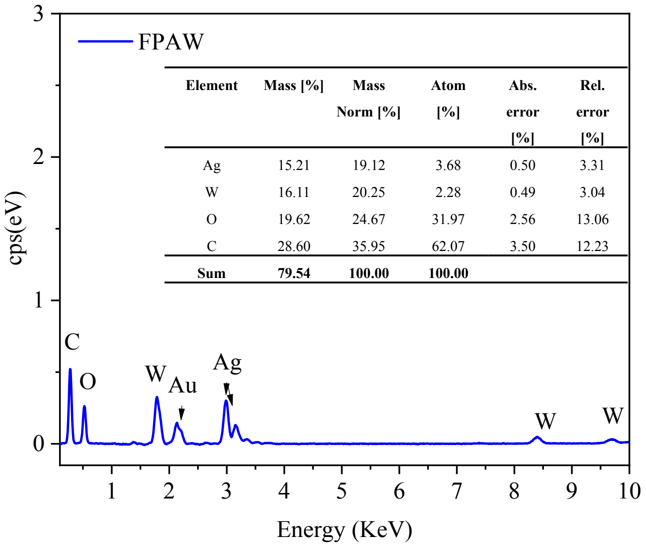

Element	Mass [%]	Mass Norm [%]	Atom [%]	Abs. error [%]	Rel. error [%]
Ag	15.21	19.12	3.68	0.50	3.31
W	16.11	20.25	2.28	0.49	3.04
O	19.62	24.67	31.97	2.56	13.06
C	28.60	35.95	62.07	3.50	12.23
**Sum**	**79.54**	**100.00**	**100.00**		

Figure S2. Spectra EDS of the FPAW polymeric mats obtained by the electrospinning method.

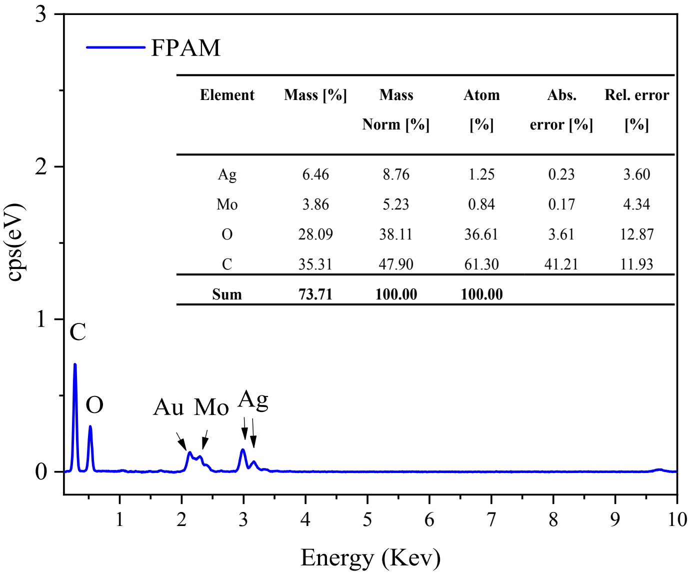

Element	Mass [%]	Mass Norm [%]	Atom [%]	Abs. error [%]	Rel. error [%]
Ag	6.46	8.76	1.25	0.23	3.60
Mo	3.86	5.23	0.84	0.17	4.34
O	28.09	38.11	36.61	3.61	12.87
C	35.31	47.90	61.30	41.21	11.93
**Sum**	**73.71**	**100.00**	**100.00**		

Figure S3. Spectra EDS of the FPAM polymeric mats obtained by the electrospinning method.

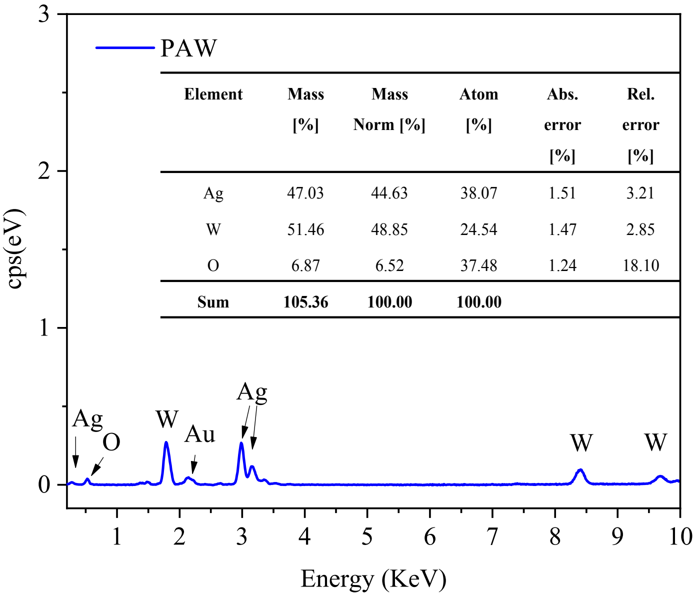

Element	Mass [%]	Mass Norm [%]	Atom [%]	Abs. error [%]	Rel. error [%]
Ag	47.03	44.63	38.07	1.51	3.21
W	51.46	48.85	24.54	1.47	2.85
O	6.87	6.52	37.48	1.24	18.10
**Sum**	**105.36**	**100.00**	**100.00**		

Figure S4. Spectra EDS of the PAW powder synthesized by the co-precipitation method.

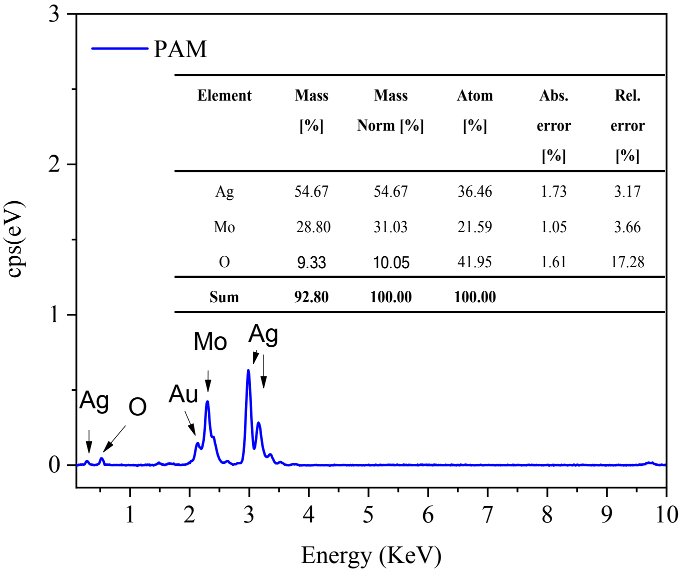

Figure S5. Spectra EDS of the PAM powder synthesized by the co-precipitation method.

3.2. Mechanical properties

The effect of PAW and PAM powders on the mechanical properties of PVA nanofibers was studied through the stress-strain curve, summarized in the Table S1. The results showed an increase in the tensile strength of the nanofibers after the addition of semiconductors, with values of 15.68 ± 0.59 MPa for FPVA, 31.47 ± 1.46 MPa for FPAM, and 42.73 ± 1.44 MPa for FPAW. The increase in tensile strength with the addition of semiconductors indicates that the material became more resistant. This may occur due to the reinforcement provided by the particles, better stress distribution, or physicochemical interactions with the polymer. This is evidenced by the elongation values of 62 ± 7.63 % for PVA, 60 ± 6.90 % for FPAM, and 46.33 ± 4.04 % for FPAW[1, 2].

Table S1 – Tensile strength (Ϭ), tensile modulus (E) and Elongation at break of FPVA, FPAM e FPAW nanofibers.

Samples	Ϭ (MPa)	Ϭ ± DP (MPa)	E ± DP (MPa)	Elongation at break (%)
FPAW1	43.43	42.73 ± 1.44	13.84 ± 0.90	46.33 ± 4.04
FPAW2	41.08			
FPAW3	43.70			
FPAM1	31.35	31.47 ± 1.46	16.17 ± 1.20	60.00 ± 6.90
FPAM2	30.08			
FPAM3	33.00			
FPVA1	16.00	15.68 ± 0.59	50.70 ± 0.70	62.00 ± 7.63

FPVA2	15.00
FPVA3	16.07

3.3 Zeta potential measures

As shown in Fig. S6(a-c), related to the polymeric mats, the zeta potentials exhibit slightly positive values, while the powders PAM and PAW, Fig.S6(d-e) show significantly more positive values. Positive zeta potential refers to the surface electric charge of suspended particles and is a measure of the electrostatic potential at the interface between a particle and the fluid in which it is suspended, indicating the magnitude and sign of the particle's surface charge.

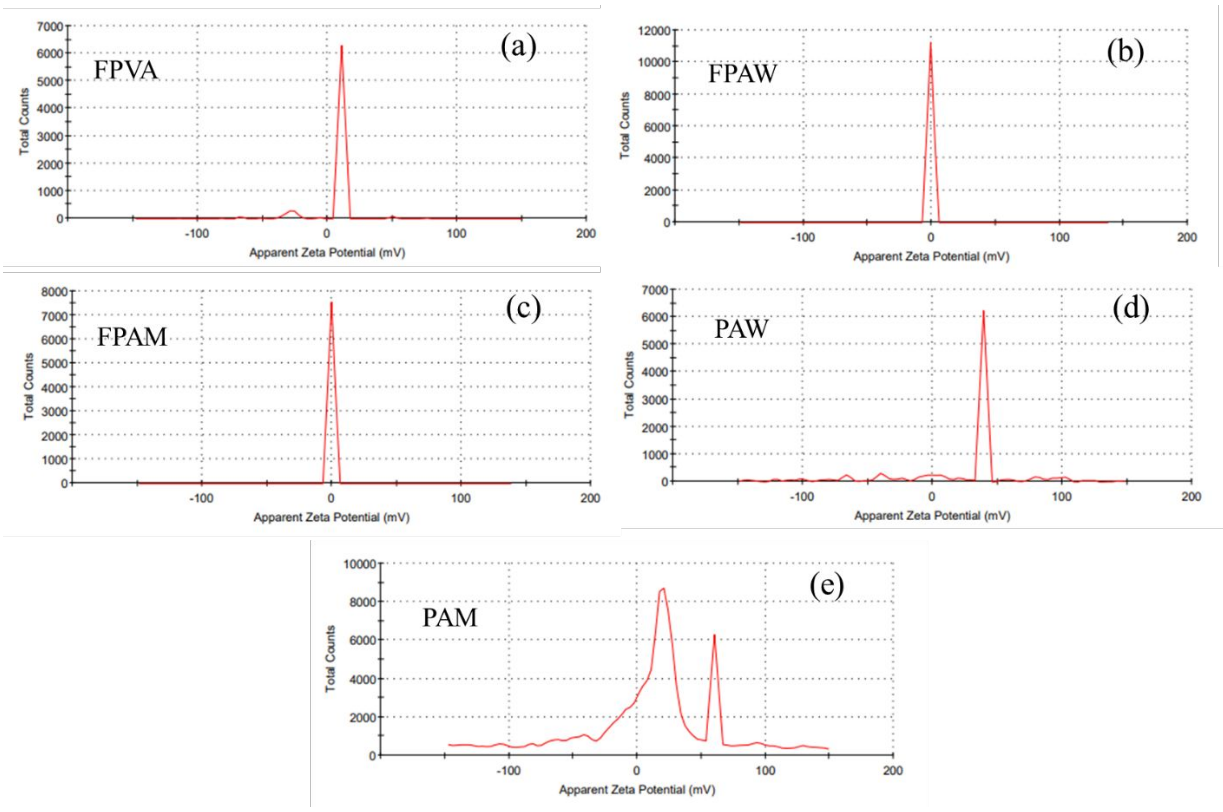

Figure S6. Zeta potential of (a) FPVA, (b) FPAW, (c) FPAM, (d) PAW and (PAM) sample.

3.4. Antimicrobial Performance

3.4.1. Minimum inhibitory concentration (MIC)

The PAW and PAM (2 mg mL^{-1}) were dispersed in sterilized distillates using an ultrasonic apparatus at 42 kHz for 25 min (25 °C), while the FPVA and FPAW and FPAM mats were in the form of discs with a diameter of 6.0 mm, having been sterilized under UV (ultraviolet) exposure for 10 min. The following parameters were adopted for the experiment:

- Negative control: sterilized water was used for the mat sample.
- Positive control: bacterial suspension.

The MIC value for the PAW sample required six successive dilutions for *Staphylococcus aureus* and two for *Pseudomonas aeruginosa*, while for the PAM sample, just one dilution was needed for both type of bacteria. For the FPVA, FPAW, and FPAM samples, 1, 3, 6, and 10 disks were added, respectively. Table S1 summarizes the digital images for the MIC determination procedure.

Table S2- Digital images for the MIC for the PAW, PAM, FPVA, FPAW and FPAM samples.

PAW sample (2 mg mL^{-1})

Staphylococcus aureus

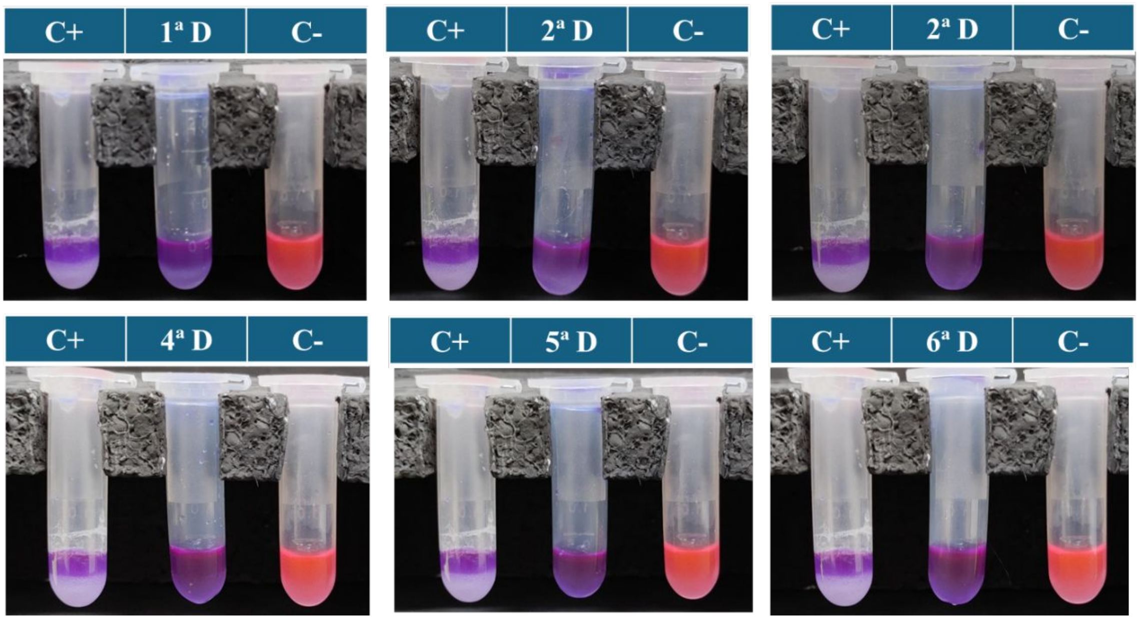

Pseudomonas aeruginosa

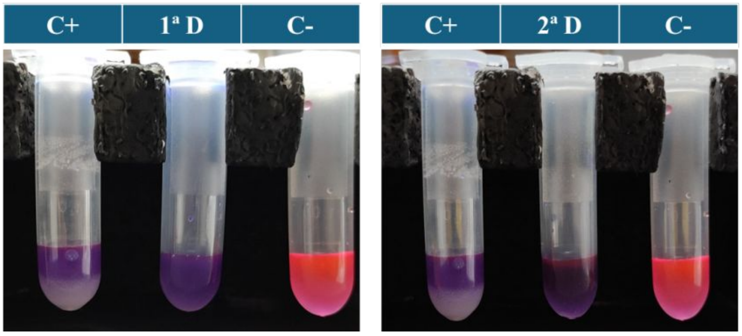

PAM sample (2 mg. mL^{-1})

Staphylococcus aureus

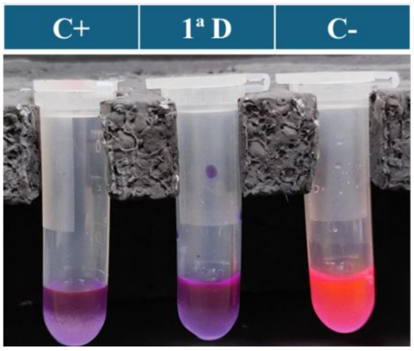

Pseudomonas aeruginosa

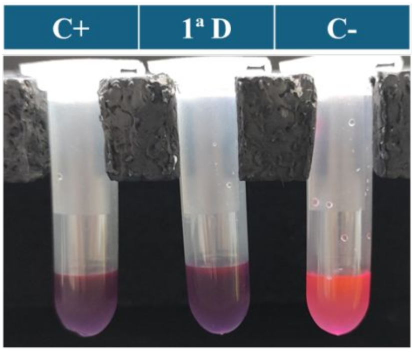

FPVA Sample

Staphylococcus aureus *Pseudomonas aeruginosa*

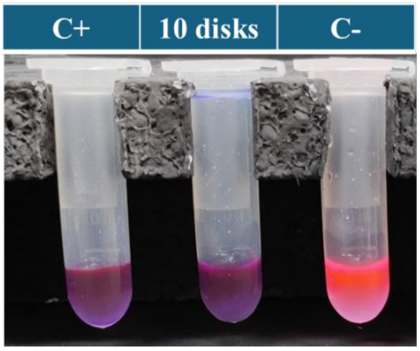

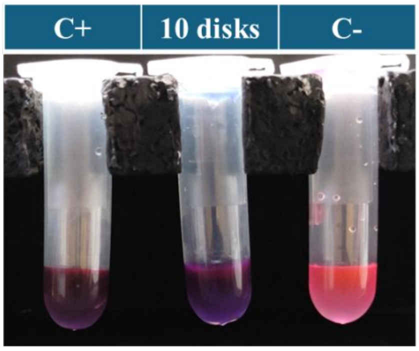

FPAW Sample

Staphylococcus aureus *Pseudomonas aeruginosa*

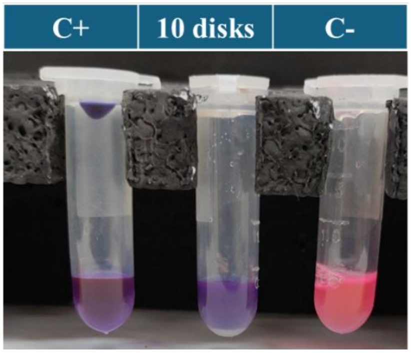

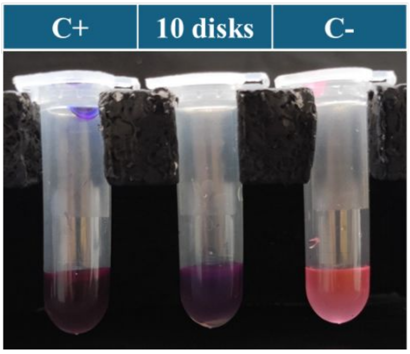

FPAW Sample

Staphylococcus aureus *Pseudomonas aeruginosa*

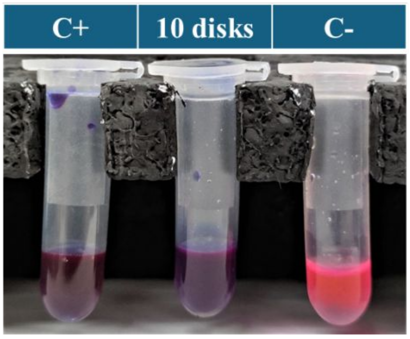

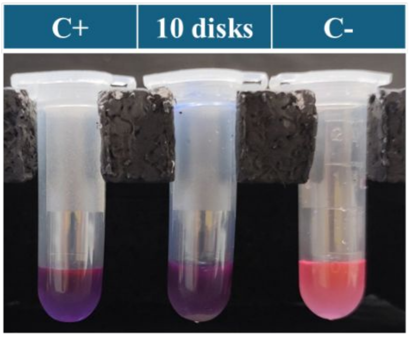

* D = dilution; **C+ = positive control, ***C- = negative control

3.4.2. Minimum bactericidal concentration (MBC)

Table S3- Digital images for the MBC for the PAW, PAM, FPVA, FPAW and FPAM samples.

FPVA Sample

Staphylococcus aureus *Pseudomonas aeruginosa*

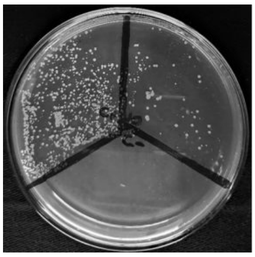
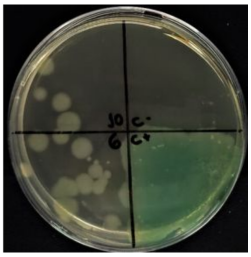

FPAW Sample

Staphylococcus aureus

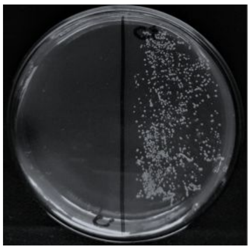
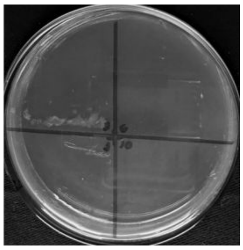

Pseudomonas aeruginosa

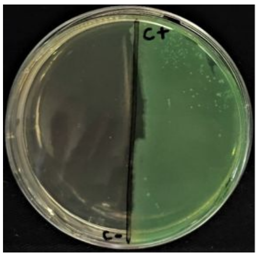
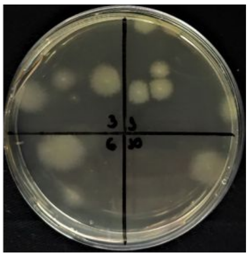

FPAM Sample

Staphylococcus aureus

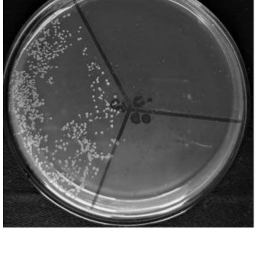
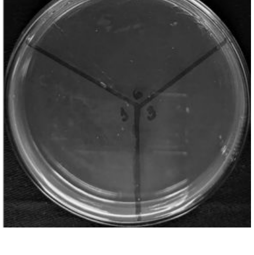

Pseudomonas aeruginosa

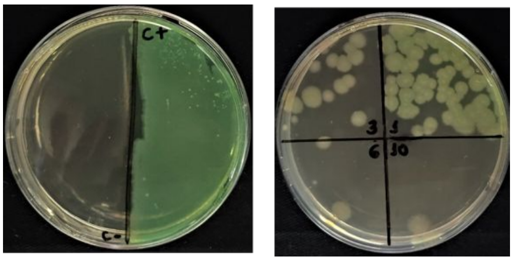

PAW Sample

Staphylococcus aureus

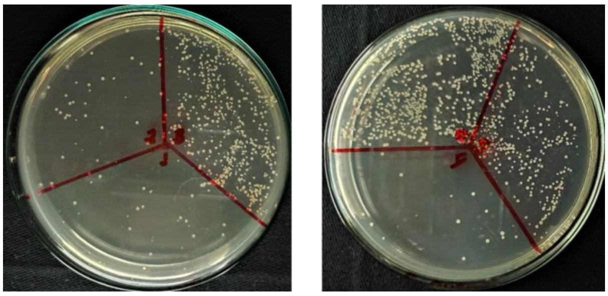

Pseudomonas aeruginosa

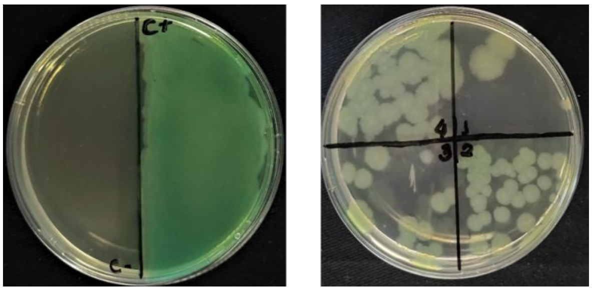

PAM Sample

Staphylococcus aureus

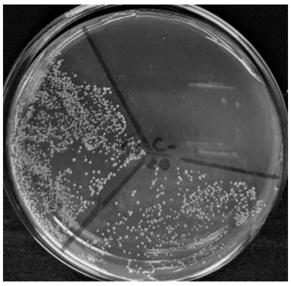

Pseudomonas aeruginosa

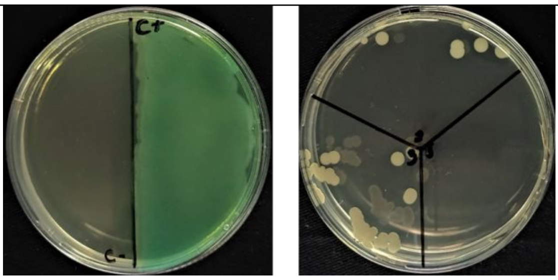

4. References

(1) Alvarado, M. Recent progress in polyvinyl alcohol (PVA)/nanocellulose composite films for packaging applications: A comprehensive review of the impact on physico-mechanical properties. *FOOD BIOENGINEERING* **2024**, *3*, 189-209. DOI: 10.1002/fbe2.12086.

(2) Khaksarfard, Y.; Ziyadi, H.; Heydari, A. Preparation of ceramic nanofibers of iron vanadate using electrospinning method. *Materials Science-Poland* **2019**, *37* (4), 645-651, Article. DOI: 10.2478/msp-2019-0070.
